# Supplementary material for: Validation of the Pathogenic Effect of IGHMBP2 Gene Mutations Based on Yeast S. cerevisiae Model
Source: Int J Mol Sci. 2022 Aug 31;23(17):9913. doi: 10.3390/ijms23179913 (PMC9456350; doi:10.3390/ijms23179913)
Supplement: Supplementary file 1 [file ijms-23-09913-s001.zip › ijms-1859622-supplementary.pdf]

Table S1. The primer sequences for site directed mutagenesis.

Mutation	Primer Forward	Primer Reverse
	IGHMBP2	
c.151C>G p.Gln51Glu	GTTTGCTGAAGCTGGAGGTATCCAGCCAG	CTGGCTGGATACCTCCAGCTTCAGCAAAC
c.163C>T p.Gln55*	CAGGTATCCAGCTAGCGCACTGGGC	GCCCAGTGCGCTAGCTGGATACCTG
c.223G>A p.Ala75Thr	CGATACGGGTCCACGGCAGCTCTTC	GAAGAGCTGCCGTGGACCCGTATCG
c.G595C p.Ala199Pro	CCTCCCAGAAAGAACCGGTTTTATTTGCGC	GCGCAAATAAAACCGGTTCTTTCTGGGAGG
c.734A>G p.Asn245Ser	GCGCCCCCTCCAGCATCGCCGTGGAC	GTCCACGGCGATGCTGGAGGGGGCGC
c.767C>G p.Ala256Gly	GTGGAGCGCCTGGGTCTGTGTAAGCAG	CTGCTTACACAGACCCAGGCGCTCCAC
c.823A>G p.Ile275Val	CTCCTGGAGTCCGTTCAGCAGCACTC	GAGTGCTGCTGAACGGACTCCAGGAG
c.1082T>C p.Leu361Pro	CCGATGGCCCCCCGAAGTTGCTGCC	GGCAGCAACTTCGGGGGGCCATCGG
c.C1478T p.Thr493Ile	CTCTTGGTGGACATCGCCGGCTGCGG	CCGCAGCCGGCGATGTCCACCAAGAG
c.G1738A p.Val580Ile	GTGATACTGTCCTTCATCAGATCCAACAGG	CCTGTTGGATCTGATGAAGGACAGTATCAC
c.C1794A p.Asn598Lys	CCGGAGGATCAAAGTGGCTGTCACC	GGTGACAGCCACTTTGATCCTCCGG
	HCS1	
c.G643C p.Ala215Pro	GAATGACTCCCAGAAAACTCCCATTAATTTTGCCATTAAC	GTTAATGGCAAAATTAATGGGAGTTTTCTGGGAGTCATTC
c.CG1574TC p.Thr525Ile	CAAAAATTCCTTTAATTTGGTATGATATCCAAGGTGATGAATTTCAAGAGACTGC	GCAGTCTCTTGAAATTCATCACCTTGGATATCATACCAAATTAAAGGAATTTTTG
c.G1846A p.Val616Ile	GAAAAAGATGTTATCATATTGAGTTTAATTCGTAGCAATGAAAAATTTGAAGTTG	CAACTTCAAATTTTTCATTGCTACGAATTAAACTCAATATGATAACATCTTTTTC
c.C1902A p.Asn634Lys	GAAGAACGAAGACTGAAAGTCGCCATGACAAGAC	GTCTTGTCATGGCGACTTTCAGTCTTCGTTCTTC

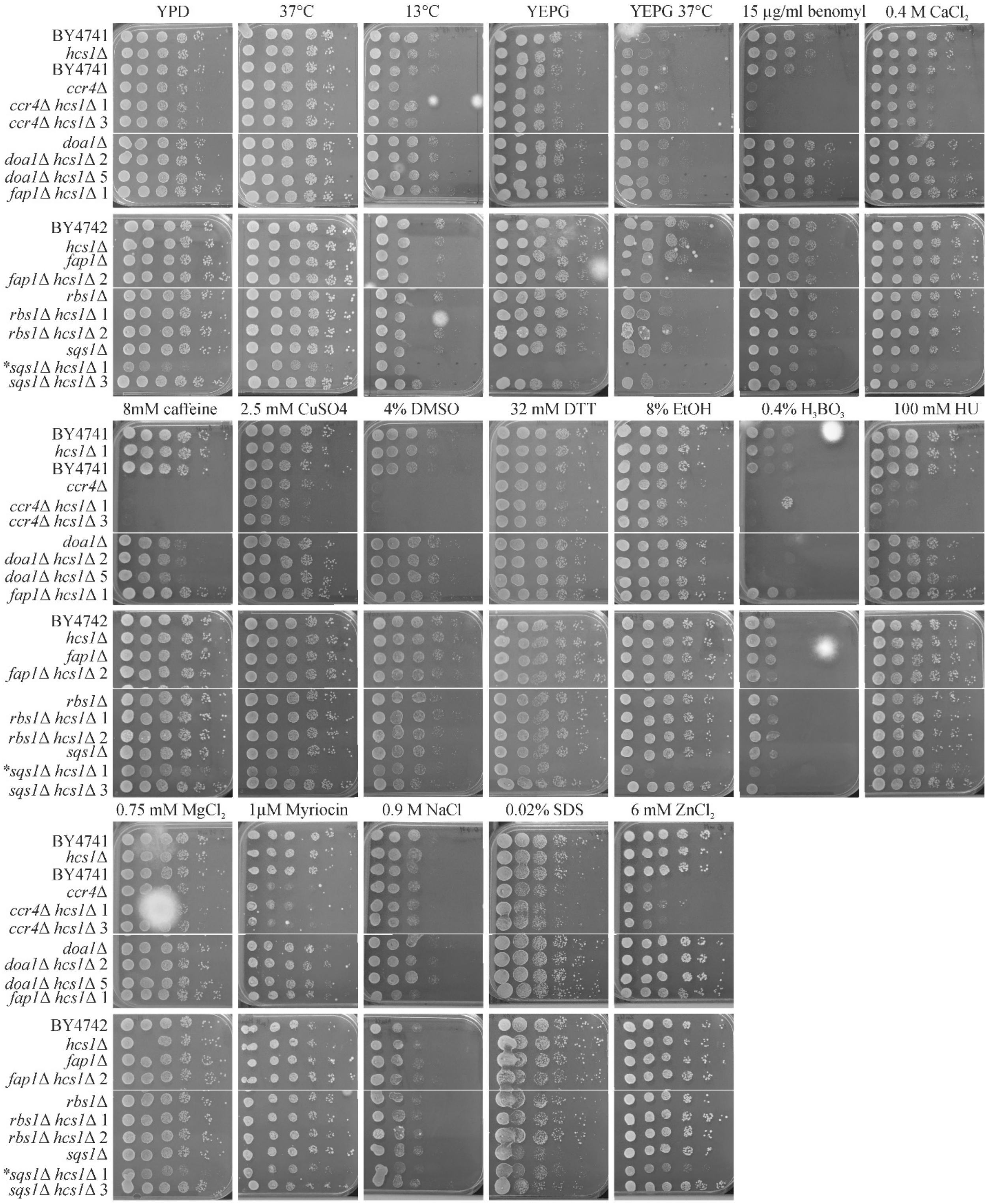
YPD
37°C
13°C
YEPG
YEPG 37°C
15 μg/ml benomyl
0.4 M CaCl2
8mM caffeine
2.5 mM CuSO4
4% DMSO
32 mM DTT
8% EtOH
0.4% H3BO3
100 mM HU
0.75 mM MgCl2
1μM Myriocin
0.9 M NaCl
0.02% SDS
6 mM ZnCl2
BY4741
hcs1Δ
hcs1Δ 1
BY4741
ccr4Δ
ccr4Δ hcs1Δ 1
ccr4Δ hcs1Δ 3
doa1Δ
doa1Δ hcs1Δ 2
doa1Δ hcs1Δ 5
fap1Δ hcs1Δ 1
BY4742
hcs1Δ
fap1Δ
fap1Δ hcs1Δ 2
rbs1Δ
rbs1Δ hcs1Δ 1
rbs1Δ hcs1Δ 2
sqs1Δ
*sqs1Δ hcs1Δ 1
sqs1Δ hcs1Δ 3

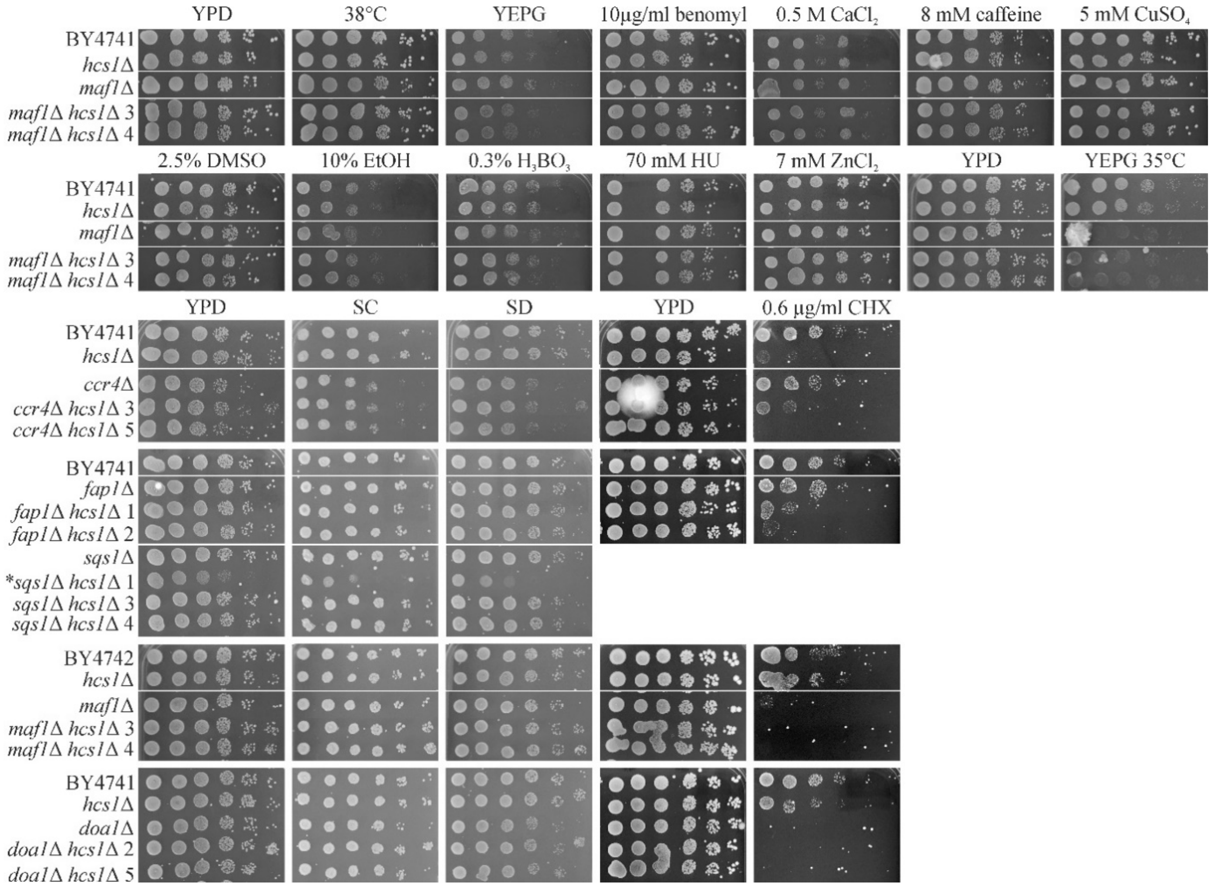

Figure S1. Growth of wild-type (BY4741 or BY4742 strain) and different mutant strains, in various conditions. The yeast cells were grown overnight in YPD medium, diluted to OD_{600} ≈1 and ten-fold serial dilutions were spotted on YPD, YEPG, SD, SC or YPD medium with indicated supplements. Respiratory deficient strains are indicated by *.

```
                      A199                                    T493
                       |                                       |
IGHMBP2  192  LDTSQKEAVLFALSQ  206     486  VPLLLVDTAGCGLFE  500
Hcs1     208  LNDSQKTAINFAINN  222     518  IPLIWYDTQGDEFQE  532
              *: *** *: **:.:                :**:  ** *  : *
                       |                                       |
                      A215                                    T525

                      V580                                    N598
                       |                                       |
IGHMBP2  573  EAVILSFVRSNRKGE  587     591  LAEDRRINVAVTRAR  605
Hcs1     609  DVIILSLVRSNEKFE  623     627  LKEERRLNVAMTRPR  641
              :.:***:****.* *                * *:**:***:**.*
                       |                                       |
                      V616                                    N634
```

Figure S2. The homology of regions containing the amino acids residues conserved between human IGHMBP2 and yeast Hcs1 proteins, which are substituted in patients (Ala199Pro, Thr493Ile, Val580Ile and Asn598Lys; in yeast respectively: Ala215Pro, Thr525Ile,Val616Ile and Asn634Lys). Alignment was prepared using Clustal Omega tool (EMBL-EBI).
